# mHealth Interventions to Improve Cancer Screening and Early Detection: Scoping Review of Reviews

**DOI:** 10.2196/36316

**Published:** 2022-08-15

**Authors:** Désirée Schliemann, Min Min Tan, Wilfred Mok Kok Hoe, Devi Mohan, Nur Aishah Taib, Michael Donnelly, Tin Tin Su

**Affiliations:** 1 Centre for Public Health Queen’s University Belfast Belfast United Kingdom; 2 South East Asia Community Observatory (SEACO) Monash University Malaysia Subang Jaya Malaysia; 3 Global Public Health Jeffrey Cheah School of Medicine and Health Sciences Monash University Malaysia Subang Jaya Malaysia; 4 Department of Surgery, Faculty of Medicine University Malaya Cancer Research Institute University of Malaya Kuala Lumpur Malaysia

**Keywords:** mobile health, mHealth, cancer screening, scoping review of reviews, cancer, cancer detection, oncology, digital health, scoping review, review, mobile phone

## Abstract

**Background:**

Cancer screening provision in resource-constrained settings tends to be opportunistic, and uptake tends to be low, leading to delayed presentation and treatment and poor survival.

**Objective:**

The aim of this study was to identify, review, map, and summarize findings from different types of literature reviews on the use of mobile health (mHealth) technologies to improve the uptake of cancer screening.

**Methods:**

The review methodology was guided by the PRISMA-ScR (Preferred Reporting Items for Systematic Reviews and Meta-Analyses extension for Scoping Reviews). Ovid MEDLINE, PyscINFO, and Embase were searched from inception to May 2021. The eligible criteria included reviews that focused on studies of interventions that used mobile phone devices to promote and deliver cancer screening and described the effectiveness or implementation of mHealth intervention outcomes. Key data fields such as study aims, types of cancer, mHealth formats, and outcomes were extracted, and the data were analyzed to address the objective of the review.

**Results:**

Our initial search identified 1981 titles, of which 12 (0.61%) reviews met the inclusion criteria (systematic reviews: n=6, 50%; scoping reviews: n=4, 33%; rapid reviews: n=1, 8%; narrative reviews: n=1, 8%). Most (57/67, 85%) of the interventions targeted breast and cervical cancer awareness and screening uptake. The most commonly used mHealth technologies for increasing cancer screening uptake were SMS text messages and telephone calls. Overall, mHealth interventions increased knowledge about screening and had high acceptance among participants. The likelihood of achieving improved uptake-related outcomes increased when interventions used >1 mode of communication (telephone reminders, physical invitation letters, and educational pamphlets) together with mHealth.

**Conclusions:**

mHealth interventions increase cancer screening uptake, although multiple modes used in combination seem to be more effective.

## Introduction

### Background

Globally, cancer is the second leading cause of death; it accounted for approximately 9.6 million deaths in 2018 [1]. Cancer incidence and mortality are predicted to increase to 30.2 million cases and 16.3 million deaths by 2040, respectively, because of aging populations and the adoption of unhealthy lifestyles [2]. Delay between symptom onset and treatment leads to poorer cancer survival [3]. Screening increases the chance of early detection and treatment and, ultimately, survival. In many high-income countries, population-based cancer screening is available for four common cancers and has contributed to reduced breast cancer [4], cervical cancer [5], prostate cancer [6], and colorectal cancer [7] mortality. However, cancer screening in the majority of low- and middle-income countries (LMICs) is opportunistic, and uptake is low compared with cancer screening in high-income countries, leading to delayed presentation, treatment, and survival [8]; for example, the uptake of mammogram screening was 12% to 31% in Brazil [9] and 7% to 25% in Malaysia [10] compared with 66% in Germany [11] and 75% in Spain [12]. Low uptake of cancer screening might indicate poor awareness and knowledge of cancer and cancer screening among the public; for example, Asian Pacific populations with the lowest uptake of colorectal cancer screening, such as India, Malaysia, Indonesia, Pakistan, and Brunei, had correspondingly low levels of awareness and knowledge of colorectal cancer symptoms, risk factors, and screening tests [13]. Poor knowledge about, and negative perceptions toward, mammogram screening are major barriers to mammogram screening uptake in Malaysia [10].

Digital health care, that is, the use of digital technologies for health, is now commonly used in public health care as well as primary health care [14]. According to the World Health Organization Global Observatory for eHealth, *mobile health* (mHealth) is defined as “medical and public health practice supported by the use of mobile devices” such as mobile phones, smartphones, and tablet computers [15]. Worldwide, there are approximately 5.3 billion unique mobile phone users, representing 67.1% of the total population, and smartphones account for approximately 75% of the mobile phones in use [16]. The high penetration rate of mobile phones allows timely data collection as well as transmission and analysis of the data. Thus, mHealth holds great potential for improving health outcomes because of its mobility, instantaneous access, and ease of use. Some of the common mHealth apps offer patient education and behavior change communication, data collection and reporting, population health registries and vital event tracking, and electronic health records, as well as provider training and education [17]. mHealth interventions have a positive impact on clinical outcomes, adherence to treatment and care, health behavior changes, disease management, and primary care attendance rates with regard to various diseases [18]. mHealth has also been used in cancer self-care and self-management among cancer survivors to improve sleep and quality of life; reduce fatigue, stress, and pain; and promote health behaviors such as weight loss [19-22]. The role of mHealth in promoting cancer screening has been explored in different types of reviews. However, it is unknown whether similar findings are observed across the reviews.

### Objectives

This scoping review aimed to map and summarize findings from systematic, scoping, narrative, and rapid reviews on the use of mHealth in cancer screening, as well as other screening-related outcomes such as attitudes toward screening and knowledge and awareness of screening. We also included implementation considerations for successful mHealth interventions in improving cancer screening uptake and screening-related outcomes.

## Methods

### Overview

This scoping review of reviews was conducted based on the framework of Arksey and O’Malley [23] and using the PRISMA-ScR (Preferred Reporting Items for Systematic Reviews and Meta-Analyses extension for Scoping Reviews) guidelines [24]. The protocol of this review has not been preregistered. As the use of mHealth in relation to cancer screening is a relatively nascent field of study, a scoping review is useful in mapping the published literature comprehensively and systematically. The review was guided by the following 5-step framework: (1) identifying the research question; (2) identifying relevant studies; (3) study selection; (4) charting the data; and (5) collating, summarizing, and reporting the results.

### Search Strategy

We first searched Ovid MEDLINE, PyscINFO, and Embase for relevant literature on February 1, 2021, using two categories of key terms: mHealth and early detection of cancer. We then refined the search on May 17, 2021. The key terms were based on Medical Subject Headings indexing as well as free-text terms. We combined key terms from the same category with OR and between categories with AND. The search strategy was developed in Ovid MEDLINE (Multimedia Appendix 1) and adapted for the other databases. We also hand searched the reference lists of selected reviews for relevant reviews. All searches were exported into EndNote (Clarivate), and duplicates were removed.

### Inclusion Criteria

Papers were included if they satisfied all of the following criteria: (1) a review of any type, (2) the reviewed interventions related to cancer screening (for any cancer type) that were conducted on mobile devices such as mobile phones and tablet computers, (3) described the effectiveness and implementation of mHealth interventions on outcomes related to cancer screening, (4) included adults aged ≥18 years from the general population, and (5) published in English in peer-reviewed journals from inception up to May 2021. We excluded reviews that did not specify the use of mobile technologies but instead reported modes of delivery such as web-based and computer-delivered programs and videos.

### Selection of Reviews

**Figure 1:**
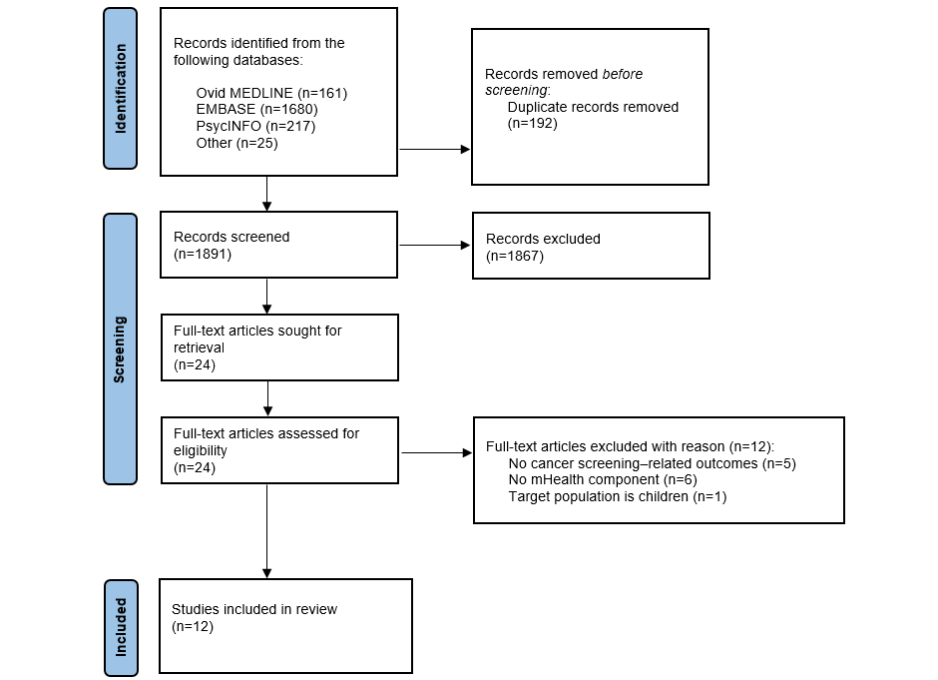
PRISMA (Preferred Reporting Items for Systematic Reviews and Meta-Analyses) flowchart.

We selected the relevant reviews using a 3-stage process: (1) MMT and WMKH conducted the initial screening of titles and abstracts to determine eligibility for inclusion; (2) WMKH retrieved full texts, which were screened by DS, MMT, and WMKH independently for inclusion, with discrepancies resolved through discussion with DS, MMT, and WMKH; and (3) MMT and WMKH extracted relevant data. The screening process is provided in the PRISMA (Preferred Reporting Items for Systematic Reviews and Meta-Analyses) flowchart (Figure 1).

### Data Extraction and Charting

The following data were extracted by MMT and WMKH from each selected review into an Excel (Microsoft Corporation) spreadsheet:

- Review identifiers (author, year, country, type of review, number of studies, time range, intervention duration, and follow-up duration)
- Study aim
- Types of cancer mentioned in the relevant studies in the review
- Types of mHealth mentioned in the relevant studies in the review
- Details of intervention procedures
- Outcome measures (awareness, knowledge, or attitude; screening uptake; and implementation-related outcomes)
- Key stakeholders in delivering the mHealth intervention, if any

If the aforementioned data were not reported in the selected reviews, we referred to the individual studies included in the selected reviews. For reviews that included studies that focused on mHealth and studies that did not, we only extracted information specifically reported on the studies that included mHealth. Information related to the quality of the reviews was not assessed.

## Results

### Literature Search

Our initial search identified 2083 citations, resulting in 1981 (95.1%) unique citations after removal of duplicates (Figure 1). The titles and abstracts were assessed based on the inclusion criteria, and of the 1981 unique citations, 24 (1.21%) were included for full-text screening. Of these 24 reviews, 12 (50%) were excluded after the full-text screen: 5 (42%) did not include cancer screening-related outcomes, 6 (50%) did not include mHealth components, and 1 (8%) included children as their target population. Hence, of the 24 reviews included for full-text screening, 12 (50%) were included in this scoping review. Table 1 summarizes the characteristics of the included reviews.

**Table 1:** Characteristics of included reviews.

Study	Type of review	Aim	Time frame of search strategy	Total number of studies; number of relevant studies^a^ by cancer type	Type of mHealth^b^ in relevant studies^a^	Key stakeholders delivering mHealth interventions
Bhochhibhoya et al [25], 2020	Scoping review	To identify studies that examined mHealth programs that focused on increasing cervical cancer screening among women to determine if these interventions improved adherence to screening and what factors (barriers and facilitators) were most influential among participants	January 1, 2009, to September, 28, 2019	12; cervical cancer (n=12, 3 of which were qualitative studies)	Telephone appointment (n=1) Telephone reminder with tailored counseling versus telephone reminder with print materials (n=1) SMS text message reminders (n=3) 15 behavior change messages with transportation e-voucher versus SMS text messages of location and hours of the closest screening clinic (n=1) Automated SMS text messages or telephone call reminders (n=1) Automated SMS text messages versus telephone call reminders+manual telephone call+face-to-face interview (n=1) 3 sequential SMS text message reminders, followed by 3 telephone call attempts (n=1)	Telephone appointment by midwife (n=1) Telephone counseling and reminders by research staff (n=1) Invitation telephone call by clinical secretaries (n=1) Telephone caller unspecified (n=1)
Uy et al [26], 2017	Systematic review	To assess the effect of SMS text messaging interventions on increasing patient adherence to screening for breast, cervical, colorectal, and lung cancers	January 2000 to January 2017	9; breast (n=5), cervical (n=1), and colorectal (n=3) cancers	SMS text message reminder (n=5) SMS text message reminder plus letter (n=4)	None
Zhang et al [27], 2020	Systematic review	To qualitatively synthesize published articles reporting the impact of mHealth on cervical cancer screening–related health behaviors	Up to October 10, 2019	8 (1 cross-sectional study); cervical cancer (n=7)	Invitation letter with pamphlet, followed by telephone reminder (n=1) Educational SMS text messages (n=2) Educational SMS text message versus SMS text message reminder (n=1) Educational SMS text messages with transportation e-voucher versus SMS text messages of location and hours of the closest screening clinic (n=1) Motivational interview over the telephone (n=1) Training through SMS text message, electronic posters, infographics, podcasts, and video tutorial (n=1)	Motivational interview through telephone call by nurses (n=1) Telephone caller unspecified (n=1)
Halake and Ogoncho [28], 2017	Scoping review	To establish the extent and nature of the published and gray literature on the use of mHealth-based technologies for cancer prevention, detection, and management in LMICs^c^	1990 to 2014	15; breast cancer (n=2)	SMS text message invitation and cancer screening information (n=1) Smartphone app to facilitate BSEd (n=1)	Not described
Choi et al [29], 2018	Systematic review	To investigate recent research trends related to the use of mobile technology in the prevention and management of skin cancer, focusing on how such technology is evaluated and what impact it has in each phase across the cancer continuum	January 1, 2007, to December 31, 2017	18; skin cancer (n=1)	Educational SMS text message about skin self-examination (n=1)	Not described
Houghton et al [30], 2019	Systematic review	To determine how mobile apps are being used for breast cancer prevention among women across the cancer control continuum	Up to February 7, 2019	69; breast cancer (n=4)	Mammopad, a decision aid mobile app on iPad Mini (n=1) Mobile app to assist navigator (n=1) mMammogram mobile app for SMS text message (n=1) Mobile app for BSE (n=1)	Mobile apps paired with community health navigators (n=2)
Plackett et al [31], 2020	Scoping review	To map the evidence for social media interventions to improve cancer screening and early diagnosis, including behavior change, and how the interventions facilitate behavior change	2004 to June 2019	23; breast (n=4) and cervical (n=1) cancers	Facebook (n=3) Snapchat (n=1)	Not described
Musa et al [32], 2017	Systematic review and meta-analysis	To review the evidence of the effectiveness of provider recommendations for cervical cancer screening on screening rates in women at risk for cervical cancer	Up to August 2016	28; cervical cancer (n=5)	Telephone counseling (n=1) SMS text message or telephone reminder (n=1) Email, telephone, or multimodal (letter+email+telephone) screening reminder and invitation and education flyer (n=1) Telephone reminder (n=1) Invitation letter and information pamphlet, followed by telephone reminder with counseling (n=1)	Telephone caller unspecified (n=3) Telephone counseling by health educator (n=1) Telemarketing company (n=1)
Duffy et al [33], 2017	Rapid review	To review the current evidence on effects of interventions to improve cancer screening participation, focusing in particular on effects in underserved populations	Time frame not specified	68; breast (n=9), cervical (n=5), colorectal (n=2), and stomach (n=1) cancers	Automated telephone and SMS text message reminders or telephone outreach (n=1) Telephone reminder or motivational telephone call (n=1) Telephone call to confirm receipt of invitation letter, followed by telephone reminder (n=1) Telephone reminders (n=8) SMS text message reminder (n=3) Tailored telephone counseling (n=2) Telephone appointment (n=1)	Colorectal cancer screening navigator (n=1) Bilingual advocate at a community organization with experience in telephone outreach (n=1) Local women recruited by Community Links, a community charity (n=1) Female scheduler and female counselors (n=1) Female research assistants (n=1) Telephone counselors (n=2) Trained GPe receptionist (n=1) Volunteers (n=1) Researcher (n=1) Research nurse (n=1) Telemarketing company (n=1) Telephone caller unspecified (n=2)
Lott et al [34], 2020	Scoping review	To map the literature on interventions to increase uptake of cervical screening in sub-Saharan Africa and identify opportunities for future intervention development and research	Up to 2019	19; cervical cancer (n=3)	SMS text message reminders (n=1) Telephone follow-up and counseling (n=1) Email (n=1)	Telephone counselors (n=1)
Déglise et al [35], 2012	Narrative review	To describe the characteristics and outcomes of SMS text messaging interventions for disease prevention in LMICs and provide recommendations for future work	Up to May 2011	17; breast cancer (n=1)	SMS text message reminder	Not described
Peiris et al [36], 2014	Systematic review	To critically appraise the role of mHealth in improving health care quality for NCDs^f^ in LMICs	Up to May 2014	48; breast cancer (n=1)	SMS text message reminder	Not described

^a^Relevant studies are studies that met the inclusion criteria for this review; for example, some reviews included diseases other than cancer. We only reported results from the studies evaluating cancer-related interventions.

^b^mHealth: mobile health.

^c^LMICs: low- and middle-income countries.

^d^BSE: breast self-examination.

^e^GP: general practitioner.

^f^NCD: noncommunicable disease.

### Characteristics of Reviews

The included reviews (n=12) were published between 2012 and 2020 (Table 1). Of the 12 reviews, 6 (50%) were systematic reviews [26,27,29,30,32,36], of which 1 (17%) also included a meta-analysis [32]; 4 (33%) were scoping reviews [25,28,31,34]; 1 (8%) was a rapid review [33]; and 1 (8%) was a narrative review [35]. The 12 reviews reported different outcomes of the studies that were relevant to this review of reviews (Table 2): 5 (42%) reported solely the effectiveness of mHealth interventions on cancer screening [26,29,32-34]; 4 (33%) reported outcomes in relation to cancer screening, change in cancer knowledge, and attitudes to screening [25,27,30,31]; 2 (17%) reported outcomes in relation to breast self-examination (BSE) practice [35,36]; and 1 reported outcomes in relation to BSE and cancer screening [28]. Most (7/12, 58%) of the reviews included studies that were conducted mainly in high-income Western countries [25,26,29-33], whereas 42% (5/12) focused on LMICs [27,28,34-36], of which 20% (1/5) focused solely on sub-Saharan Africa [34]. In total, 33% (4/12) of the reviews focused on cervical cancer [25,27,32,34]; 8% (1/12) focused on skin cancer [29]; 8% (1/12) focused on breast cancer [30]; 8% (1/12) examined breast, cervical, lung, and colorectal cancers [26]; 25% (3/12) included any type of cancer [28,31,33]; and 2 reviews focused on disease prevention in general [35,36]. In terms of interventions, 42% (5/12) of the reviews included interventions of various types of mHealth technologies [25,27-29,36], 2 (33%) focused solely on SMS text messages [26,35], 1 (17%) focused on social media interventions [31], 1 (17%) was specifically about mobile apps [30], and 25% (3/12) included any type of communication (mHealth, face-to-face, and other media) [32-34].

**Table 2:** Summary of screening-related outcomes extracted from each review.

Study	Outcomes		
	Screening outcomes	Screening awareness-, knowledge-, and attitude-related outcomes	Implementation-related outcomes and measures
Bhochhibhoya et al [25], 2020	Screening uptake: n=5/6a (9.1%-17.9% increase between intervention group versus control group; 9.3% increase after the intervention compared with before)b Screening follow-up adherence: n=0/1 Effective methods: stepwise approach (automated telephone calls and SMS text messages, followed by manual telephone call and face-to-face interview), SMS text messages only, telephone call only, telephone appointment by midwives, telephone reminders combined with other methods such as tailored counseling, and SMS text message with transportation e-voucher	Knowledge improvement: n=2/2 Attitude about screening: n=1/2 Perceived behavior control: n=0/1 Perceived barriers about screening: n=0/1 Belief about screening: n=1/1 Screening intention: n=0/2 Effective methods: health-specific and spiritually based SMS text messages and personally tailored texts with statistical facts	Advantages: convenient, time effective, ease of use, and able to receive notification Concerns: confidentiality of SMS text messages, loss of the mobile phones, clarity of the language used, and receiving negative results through SMS text message Barriers: inconvenient for older participants, lack of texting proficiency, difficulty in texting, and apprehension that SMS text messages might not be clearly understood Enabling factors: contact preferences, cell phone ownership, and portability of same number Enhancing factors: message content (reminder and informative) and short and simple messaging formats
Uy et al [26], 2017	Screening uptake: n=5/9 (1.2%-9.9% absolute increase) Effective methods: SMS text message reminder+letter and single SMS text message reminder	—^c^	—
Zhang et al [27], 2020	Screening uptake: n=3/5 (12.9%-50.9% increase) Screening follow-up: n=1/1 (91.8%-93.5%; OR^d^ 1.37-1.40) Effective methods: SMS text message with transportation e-voucher, invitation letter with telephone reminder, reminders sent through letter, registered letter, SMS text message or telephone call, and telephone reminders or educational telephone call	Knowledge improvement: n=1/2 Perceived benefits of Pap^e^ test: n=1/1 Reduced barriers to undergoing Pap smear: n=1/1 Attitude about screening: n=0/1 Effective methods: a combination of SMS text message, electronic posters, infographics, podcasts, and video tutorials	Interest in receiving screening test results through SMS text message: n=0/1 Interest in receiving screening test results using nonprivate telephone: n=1/1 (OR 0.31, 95% CI 0.18-0.51) Interest in receiving appointment reminders through SMS text message: n=1/1 (OR 14.19, 95% CI 1.72-117.13) Interest in receiving appointment reminders using nonprivate telephone: n=0/1
Halake and Ogoncho [28], 2017	Screening uptake: n=1/1 (30.7% and 31.6% increase) BSE^f^ practice: n=1/1 Effective method: BSE smartphone app	—	—
Choi et al [29], 2018	Screening uptake: n=1/1 (27% absolute increase in skin self-examination) Effective method: educational SMS text messages with reminders	—	—
Houghton et al [30], 2019	Screening uptake: n=3/3 Effective methods: community health workers (trained or untrained in patient navigation) equipped with smartphone app plus standard risk counselling and mMammogram (SMS text messages plus health navigator)	Knowledge improvement: n=2/2 (33% increase) Reduced decisional conflict: n=1/1 Self-efficacy: n=1/1 Screening intention: n=0/1 Screening readiness: n=1/1 Effective methods: smartphone app plus standard risk counseling, mMammogram (SMS text messages plus health navigator), and smartphone app decision aid (Mammopad)	Intervention satisfaction (mMammogram): n=1/1 Effectiveness satisfaction (mMammogram): n=1/1
Plackett et al [31], 2020	Screening uptake: n=1/1 (12.9% increase) Effective method: breast cancer screening service Facebook page	Knowledge improvement: n=2/2 Screening intention: n=1/1 (82% increase) Effective methods: Facebook or face-to-face discussions for 2 weeks after 50-minute classroom cervical cancer prevention education lecture (female high school students), receiving breast cancer awareness information through Snapchat, and tailored SMS text message mammography campaign on Facebook during Breast Cancer Awareness Month	Using Facebook is acceptable for delivering breast cancer screening information: n=1/1
Musa et al [32], 2017	Screening uptake: n=5/6 (7.8%-31.1% absolute increase) Reduced screening median time: n=1/1 Effective methods: direct invitation mail+brochure+telephone counseling by health educators; telephone reminder with educational information and multimodal intervention; invitation letter and information pamphlet, followed by telephone reminder with counseling; telephone reminder with educational information; and multimodal intervention	—	—
Duffy et al [33], 2017	Screening uptake: n=13/16 (5%-45% absolute increase) n=3/3, SMS text reminder studies; n=11/13, telephone reminder studies	—	—
Lott et al [34], 2020	Screening uptake: n=2/3 (8.6% difference in screening uptake between control and intervention groups; 51% increase after the intervention) Effective methods: SMS text message about cervical cancer and context-specific barriers to screening (and SMS text message plus e-voucher for transportation) and enhanced patient-centered counseling with patient follow-up by telephone (with or without escort to cervical cancer screening)	—	—
Déglise et al [35], 2012	BSE practice: n=1/1 Effective method: SMS text message reminder to conduct BSE	—	—
Peiris et al [36], 2014	BSE practice: n=1/1 Effective method: SMS text message reminder to conduct BSE	—	—

^a^Number of studies that reported a positive outcome out of the total number of studies that included the particular outcome.

^b^Percentage of change or odds ratios are included if available.

^c^Not available (ie, not reported).

^d^OR: odds ratio.

^e^Pap: Papanicolaou.

^f^BSE: breast self-examination.

### Types of mHealth Interventions

SMS text messages were the most commonly used mHealth technology and were used in 46% (31/67) of the interventions. They were mainly delivered as reminders of cancer screening appointments, alone or in combination with telephone reminders, physical invitation letters, and educational pamphlets. Educational SMS text messages, sent as a one-off or in a series over days or weeks, were also widely used. Their contents included information about cancer risk factors, benefits of screening, location and operating hours of screening clinics, spiritually based health messages, and facts about cancer (eg, incidence, mortality, and screening rates). Educational SMS text messages were used alone or in combination with an e-voucher (to subsidize the cost of transportation to and from the screening facility) [37].

Text messages were most commonly sent as SMS text messages. In later studies, they were also sent through IP-based messaging services such as Telegram and Snapchat and mobile apps specifically designed for the interventions. In almost all (10/12, 83%) reviews, the delivery of SMS text messages was one-way, 8% (1/12) of the reviews reported an intervention that included a specifically designed mobile app (mMammogram) that featured personally tailored messages [38], and 8% (1/12) used social media for communication [31].

Telephone calls were used in 40% (27/67) of the interventions mostly as cancer screening invitations and reminders and to arrange screening appointments. Telephone reminders, automated or live, were used alone or with SMS text message reminders, screening invitation letters, and pamphlets. Participants were contacted through telephone to confirm the receipt of a screening invitation letter. Motivational interviews were conducted over the telephone to increase participants’ readiness to attend screening [27]. Knowledge about cancer was provided and barriers to screening addressed through telephone counseling [25,33,34].

A few breast cancer mobile apps were specifically designed for interventions. Mammopad, for example, is a decision aid, a tool that helps women to decide to participate in mammogram screening, that ran on the iPad Mini [30]. Another app was designed to assist community health workers (CHWs) in interviewing participants, reporting data, showing a motivational video, and offering a mammogram appointment for women with an abnormal clinical breast examination (CBE). A BSE-facilitating smartphone app included BSE date reminders and a reminder to encourage mother and daughter to practice BSE together [39].

Other mHealth platforms that were less frequently used were emails and social media. Emails were used to deliver screening invitations, reminders, web-based educational flyers, and cancer- and health-related information. Social media platforms such as Facebook and Snapchat were used as intervention modes to provide information about breast and cervical cancers and screening, promote mammogram screening, and schedule breast screening appointments, as well as a platform for discussions about cervical cancer after a lecture [31].

Almost all (11/12, 92%) reviews described mHealth interventions that included 1 or 2 mHealth technologies. There was only 1 intervention that used a combination of >2 types of mHealth technologies: a training in cervical cancer through SMS text message, electronic posters, infographics, podcasts, and video tutorials [40].

### Key Stakeholders in mHealth Interventions

Of the 12 reviews, 5 (42%) included telephone call interventions that were delivered by a broad range of personnel [25,27,32-34]. Telephone reminders or telephone calls to make or confirm screening appointments were delivered by bilingual advocates from a community organization, local women recruited from a community charity, research assistants, general practitioner receptionist, volunteers, research nurses, midwives at antenatal health clinics, clinical secretaries, and telemarketers.

Among the important personnel in mHealth interventions were telephone counselors who called the participants to inquire about their screening intention and ascertain whether they had received the invitation letters, provided information about screening, addressed current or potential barriers to screening uptake through motivational interviews and applied a counseling approach to increase motivation for behavior change, or assisted with appointment scheduling. Telephone counseling was delivered by nurses or hospital-based health counselors.

Health navigator services were mentioned in 8% (1/12) of the reviews [30]. Health navigators used mobile apps to facilitate interviews, report data, show motivational videos, and offer screening appointments. Health navigators or CHWs guided participants in navigating cancer screening information, provided transportation and interpretation services, addressed technical problems related to mobile app use, and reminded participants to complete cancer screening.

### Cancer Screening Uptake

All (12/12, 100%) reviews included in this review reported mainly improved cancer screening uptake or self-examination practice (for breast or skin cancer; Table 2). The increase in screening between the intervention and control groups (from relevant studies) ranged from 1.2% to 50.9%.

Overall, the reviews concluded that interventions that included >1 communication mode seemed more effective than those that included a single telephone call or SMS text message reminder. A 3-step sequential approach (an automated reminder telephone call and SMS text message, followed by manual telephone calls and face-to-face interviews) conducted at Portuguese primary health care units resulted in 51% of the women in the intervention group attending cervical cancer screening compared with 34% of the women in the control group who received only written invitation letters [25,41]. In another study, women in northern Tanzania who received transportation e-vouchers to cover return transportation to the nearest screening facility as well as a series of 15 behavior change messages delivered through SMS text message were more likely to attend cervical cancer screening (uptake: 18%; OR 4.7, 95% CI 2.9-7.4) compared with those who received only the same SMS text message (uptake: 12.9%; OR 3.0, 95% CI 1.5-6.2) and those who received 3 SMS text messages with the location and hours of the nearest screening clinic (uptake: 4.3%) [27,34,37]. Participants from Iran who received a Health Belief Model–based training in cervical cancer through SMS text messages, electronic posters, infographics, podcasts, and a video tutorial were more likely to complete a Papanicolaou (Pap) test (47.9%) than the participants in the control group (5.8%) [27,40].

A once-a-month SMS text message reminder over 6 months combined with a BSE training through a lecture, video, and demonstration of the technique on a breast model led to a 32% increase in BSE practice [35,36,42]. An Android operating system–based smartphone app that included a BSE date alarm, a reminder to encourage mother and daughter to practice BSE together, a *mother motivation function* that allows the user to call her mother using a notification function to practice BSE together, and educational videos increased the percentage of Korean women practicing BSE from 62.2% to 71.1% [39].

Of the 12 reviews, 1 (8%) included interventions that incorporated navigation to health services [30], which was found to be effective in increasing screening uptake. All (3/3, 100%) of the interventions that included health navigation services were effective in increasing screening uptake. Korean American immigrant women who received a series of 8 to 21 SMS text messages about breast cancer through a specially designed mobile app (mMammogram) and were provided with health navigation services had a significantly higher percentage of completed mammograms after 6 months than women who received printed brochures only (75% vs 30%; *P*<.001) [30,38]. CHWs in Bangladesh who used mobile apps to facilitate CBE, such as showing a motivational video and offering an appointment, detected 3 times more women with abnormal CBEs than CHWs without smartphone support (3.1% without navigation training and 3.2% with navigation training vs 1% without smartphone) [30,43]. CHWs who used mobile apps and were trained in navigation had the highest percentage of participants with an abnormal CBE who attended further clinical assessment compared with those who used mobile apps only or without smartphone support. In a study in the United States, participants who failed to complete a fecal occult blood test were much more likely to complete a second fecal occult blood test than those in usual care if they had been contacted through telephone call by colorectal screening navigators (82.2% vs 37.3% among those who received standard care; *P*<.001) [33,44].

There were a number (46/67, 69%) of studies that used only 1 mode of mHealth communication, and the findings related to screening uptake after the intervention compared with before the intervention were mixed; for example, in an email intervention study, whether an email message was loss-framed (focused on risk), gain-framed (focused on health and well-being improvement), or neutrally framed (provided only facts) had no effect on cervical cancer screening uptake [34,45]. An exception was a study conducted in western Sweden where there was telephone contact through midwives to offer an appointment for a Pap test, which increased the uptake of Pap tests compared with the usual annual invitations without telephone contact (uptake at 3-month follow-up: 13% vs 3.9%; risk ratio 3.37, 95% CI 2.83-4.01) [25,46]. Another exception was the use of Facebook to share breast cancer information and schedule breast screening appointments, which increased breast cancer screening attendance by an average of 12.9% [31,47].

A brief invitation SMS text message was as effective as a detailed informative SMS text message: there was no significant difference in screening uptake between Lebanese women who received an SMS text message mammogram invitation and those who received the same SMS text message and an additional informative SMS text message about the benefits of mammogram screening [28,48].

### Screening Awareness, Knowledge, Intention, and Attitude

Of the 12 reviews, 4 (33%) [25,27,30,31] included studies specifically on knowledge, awareness, intention, or attitude in relation to cervical cancer screening (2/4, 50%), breast cancer screening (1/4, 25%), or both (1/4, 25%), and almost all of the individual interventions (7/8, 88%) reported improvements in knowledge, whereas few studies reported an improvement in screening intention (1/4, 25%; Table 2). Interventions that were successful in increasing screening uptake were also successful in increasing knowledge and awareness about screening for both cervical and breast cancer.

The CervixCheck intervention was designed for African American women and consisted of a series of 22 health-specific, spiritually based, cervical cancer–related SMS text messages (eg, on the importance of keeping the body healthy and attending screening) that were sent over 16 days. It resulted in a significant increase in knowledge about cervical cancer and the Pap test (mean difference=0.619; *P*=.001) [25,49]. A 1-week personally tailored SMS text message intervention significantly increased Korean American women’s knowledge of cervical cancer screening guidelines (mean difference=0.31-0.71; *P*=.006) [25,50]. Participants who went through the Health Belief Model–based cervical cancer training scored significantly higher in perceived benefits of a Pap test and lower in barriers to obtaining a Pap test, in addition to a higher uptake of Pap tests [27,40]. Female high school students who participated in Facebook or face-to-face discussions for 2 weeks after a 50-minute classroom cervical cancer prevention education lecture that included knowledge about Pap testing increased their knowledge about cervical cancer compared with those in the control group (β=2.942; *P*<.001) [31,51]. Compared with a telephone reminder and invitation intervention, an educational telephone call that provided a brief explanation on cervical cancer, its risks, and colpocytological examination increased knowledge about colpocytological examination but not attitude toward it [27,52].

Korean women who used the mMammogram app and were provided with health navigation services had increased knowledge of breast cancer screening compared with the control group (group difference=mean 16.93, SD 4.77; *P*=.001) [30,38]. Users of Mammopad, a decision aid for mammogram screening, reduced decisional conflict and increased self-efficacy in relation to mammography, although there was no significant change in screening intention [30,53]. Saudi Arabian women who received breast cancer awareness information through Snapchat had better breast cancer awareness and knowledge, including knowledge about breast cancer screening (*P*=.01), than those in the control group who did not receive any awareness information [31,54]. Among women who were surveyed in the tailored SMS text message mammography campaign on Facebook during Breast Cancer Awareness Month, 82% intended to get a mammography in the next year [31,55].

### Implementation Outcomes and Measures

Of the 12 reviews, 4 (33%) [25,27,30,31] included studies that examined outcomes related to the implementation of mHealth in cancer screening uptake interventions (Table 2). Of these 4 reviews, 3 (75%) reported a high acceptance of such interventions [25,30,31]. In a 1-week personally tailored SMS text message intervention, 83% of the participants expressed satisfaction with the intervention, and 97% reported that they would recommend the program to their friends [25,50]. In the CervixCheck intervention, 83% of the participants reported being either “satisfied” or “very satisfied,” and 85% found the SMS text messages either “useful” or “very useful” [25,49]. The mMammogram intervention participants were satisfied with the intervention (*P*=.003) and agreed that it was effective (*P*<.001) [30,38]. In a tailored SMS text message mammography campaign on Facebook during Breast Cancer Awareness Month, 25% of the women surveyed agreed that they used Facebook to find breast cancer screening information, and 43% agreed with seeing more mammogram information on Facebook [31,55].

Some of the concerns of the participants regarding mHealth interventions included confidentiality of SMS text messages, loss of mobile phones, clarity of the language used, and receiving negative results through SMS text messages. Participants were interested in receiving SMS text message reminders for appointments; however, there was reluctance to receive screening results through SMS text messages in case someone else accessed their mobile phones and saw the results (OR 0.31, 95% CI 0.18-0.51), although they reported no issue with making an appointment.

The barriers to using mHealth in reaching out to people to encourage cancer screening included inconvenience for older participants, lack of texting proficiency, difficulty in texting, and apprehension that SMS text messages might not be clearly understood [56]. Including a reminder and keeping the SMS text messages informative, short, and simple was suggested to increase screening uptake [57].

## Discussion

### Principal Findings

This scoping review of reviews suggests that mHealth interventions can be effective in increasing cancer screening uptake and practice, as well as improving other screening-related outcomes such as knowledge and awareness about screening. The results are consistent across different types of reviews. The most commonly used mHealth technologies used were SMS text messages and telephone calls. Interventions that included >1 mode of communication, such as telephone calls and SMS text message reminders combined or together with invitation letters, health education, or navigation services, seemed to be more effective than interventions that included only 1 mode of communication. A few (4/12, 33.3%) of the reviews reported implementation measures, and 75% (3/4) suggested that mHealth interventions were well accepted by participants.

The effectiveness of interventions that used >1 mode of communication has been demonstrated in cancer screening uptake in LMICs [58]; for example, in Malaysia, mass media campaigns that used different channels of health promotion successfully increased symptom awareness of breast cancer [59] and colorectal cancer [60].

A very effective intervention was a combination of educational SMS text messages and e-vouchers to subsidize the transportation to attend screening [37], which is especially relevant in rural areas in LMICs. In many LMICs, public transport and e-hailing services are mainly available in cities, and the majority of health care facilities that offer cancer screening are located in town areas; for example, in Malaysia, travel distance to the nearest mammogram screening facility ranged between 2 km and 340 km with a median of 22 (IQR 12-42) km [61]. Longer travel distance to cancer services is associated with lower likelihood of cancer screening uptake [62] and presentation of more advanced stages of breast cancer [63] and colorectal cancer [64]. Interventions that increase knowledge might not translate into higher screening uptake if underlying structural barriers to screening, such as lack of transportation, are not addressed [25,27]. The use of e-vouchers has been described as a form of an “enablement” intervention that reduces “barriers to increase capability or opportunity” [65].

Approximately half (31/67, 46%) of the interventions included in the reviews included SMS text messaging, which uses a cellular network and is preinstalled on every mobile phone, unlike internet-based instant messaging apps. Almost 100% of SMS text messages are read, and 90% of them are read within 30 minutes of receipt compared with emails (approximately 18% are read) [66], which might explain the ineffectiveness of emails in improving cancer screening uptake and related outcomes. Worldwide, IP-based chat apps are gaining popularity: WhatsApp, Facebook Messenger, and WeChat have 2 billion users, 1.3 billion users, and 1.2 billion users, respectively [67]. Chat apps, especially those with high open rates, such as SMS text messaging [68], enable more efficient communication by allowing users to send longer messages; share pictures, videos, or audio messages; and chat in real time. However, because SMS text messaging is operator-based, it is more useful in rural areas where there is poor mobile internet coverage. In addition, SMS text messaging is simple to use and does not require additional apps, which might be more user friendly for those who are less tech savvy; for example, older adults.

In addition to SMS text messaging and chat apps, social networking sites, with their large numbers of users, hold great potential in mHealth interventions. As of July 2021, popular social networking sites such as Facebook and Instagram had 2.85 billion users and 1.39 billion users, respectively, and the numbers are increasing rapidly [67]. However, in the only review that examined social media solely [31], the studies included were mostly about low-level engagement (number of impressions, reach, likes, comments, and sharing of tweets and posts), and the review highlighted the lack of studies (1/4, 25%) that examined high-level engagement with social media interventions, such as uptake of screening [31]. This is likely because of the difficulty in linking screening uptake and social media data because social media posts are not designed for such analysis. The fast pace of social media means that social media contents could be outdated quickly or get inundated by other information, which reduces their reach to the target population and long-term sustainability. Running multiple campaigns on multiple social media platforms also means that it is difficult to pinpoint which campaign or platform has the greatest impact on behavior change. In addition, there are age differences in social media use; many individuals in the targeted age groups for cancer screening might not be reached through social media. In a survey of American adults, >80% of those aged 18 to 49 years and 73% of those aged 50 to 64 years used social media sites, whereas only 49% of those aged ≥65 years reported so [69].

mHealth interventions will only work if there is access to mobile phones and mobile internet. Globally, although the penetration of mobile phones and mobile internet is high, there is an unequal access to mobile technology and internet between urban and rural areas and between sexes. All urban areas are covered by a mobile broadband network; however, in some LMICs, 19% of the rural population are covered by only a second generation network, and 17% of the rural population have no mobile coverage at all [70]. The rural-urban gap is especially prominent in LMICs, where urban access to a mobile broadband network is 2.3 times as high as rural access [70]. In LMICs, women’s mobile phone ownership and internet use is significantly lower than that of men’s, and the gap ranges from 50% in South Asia and 20% in sub-Saharan Africa to 12% in the Middle East and North Africa [71].

The gap in mobile phone ownership and internet use has important public health implications. mHealth interventions to increase cancer screening uptake might be less effective in rural areas, where screening uptake is already low [72]. Many (57/67, 85%) of the mHealth interventions targeted cervical and breast cancers, the 2 most common cancers among women. Thus, the rural-urban gap in mobile access means that women from rural areas are at a greater risk of inequitable access to information and interventions on cancer screening.

Given the rapid development of mHealth technologies, there is a need for researchers to incorporate them effectively into interventions. However, the speed of research does not advance at the speed of mobile technology, and researchers have little control over app development [30]. Most smartphone apps address tertiary cancer prevention [30], such as support for patients with cancer in health information management [73], medication adherence [74], weight management [75], and mental health improvement among cancer survivors [76], and there is a lack of smartphone apps for secondary cancer prevention. Many of the apps developed for research are not available for download and have not been widely adopted after the studies were concluded.

mHealth holds great potential to reach out to many people in low-cost settings, and it is also safe in times of the COVID-19 pandemic where social contact has to be minimized. However, it might not be acceptable to pass on personal information through certain mHealth technology; for example, there were participants who mentioned that although it was acceptable to receive SMS text message reminders about their screening appointment, they would not want to be informed about their screening results through SMS text messages. The gap could be filled by CHWs, who could act as the link between mHealth technologies and participants by informing the latter personally through telephone calls of their screening results. A recent review found that CHWs play a critical role, particularly during pandemics, in community engagement [77]. CHWs are usually members from the same communities as the intervention participants and are knowledgeable about the resources available within the communities. They may be able to reach out to vulnerable populations and encourage uptake of cancer screening [78] and mobile technologies [79]. However, despite proven effectiveness of CHWs in cancer screening and early diagnosis interventions [80], there were very few (1/12, 8%) reviews that included interventions that combined mHealth and CHWs.

### Limitations

Given the heterogeneity of reporting and differences in the details reported in each review, it was challenging to summarize the evidence from the reviews concisely. In addition, some reviews did not exclusively examine mHealth and cancer screening; they included other types of interventions and preventive measures. Furthermore, unlike in a systematic review, the quality of the selected articles was not assessed.

### Future Research and Recommendations

Future interventions should consider combining at least two modes of mHealth communication, for example, SMS text messages and telephone calls, and screening interventions are likely to achieve better attendance when participants receive at least one reminder. In addition, future interventions should consider incorporating instant messaging apps such as WhatsApp, Facebook Messenger, and WeChat, in addition to SMS text messaging, because the number of users is increasing exponentially, and more educational information using videos, audio messages, or graphics could be shared. Social media platforms, especially Facebook, should be incorporated for health promotion, sharing of educational information, and appointment making. When social media platforms are used, there is a need to take into account their popularity and acceptability within the country where the interventions are conducted. In addition, engagement with different social media apps varies among age groups. Other incentives such as transport vouchers may be included when interventions are conducted among those with poor access to screening facilities. Facilitators to improving access to, and engagement with, mHealth among older adults have been described, including support from the government and family, addressing digital problems in deprived areas, and increasing accessibility to mobile phones or tablet computers [81]. CHWs and navigation services may be provided along with mobile technologies to support participants’ needs, promote and facilitate the use of mHealth, and pass on information such as screening results.

### Conclusions

mHealth interventions have the potential to increase cancer screening uptake and other cancer screening–related outcomes such as knowledge about screening and intention to screen. Combining >1 mode of communication may have a better impact on cancer screening uptake.

## Appendix

Multimedia Appendix 1 Search strategy.

## Abbreviations

BSE: breast self-examination
CBE: clinical breast examination
CHW: community health worker
LMICs: low- and middle-income countries
mHealth: mobile health
Pap: Papanicolaou
PRISMA: Preferred Reporting Items for Systematic Reviews and Meta-Analyses
PRISMA-ScR: Preferred Reporting Items for Systematic Reviews and Meta-Analyses extension for Scoping Reviews
