# Supplementary material for: mHealth Interventions to Improve Cancer Screening and Early Detection: Scoping Review of Reviews
Source: J Med Internet Res. 2022 Aug 15;24(8):e36316. doi: 10.2196/36316 (PMC9425170; doi:10.2196/36316)
Supplement: Multimedia Appendix 1 [file jmir_v24i8e36316_app1.docx]

**Search strategy**

*Ovid MEDLINE/PsychInfo/EMBASE*

(Telemedicine/ OR (mHealth or digital intervention or mobile health or e-health or telemedicine or telehealth or text message or short message service or phone or app).mp. [mp=title, abstract, heading word, drug trade name, original title, device manufacturer, drug manufacturer, device trade name, keyword, floating subheading word, candidate term word] OR Cell Phone/ or Smartphone/) AND ("Early Detection of Cancer"/ OR (early diagnosis or early detection of cancer or mass screening or low-dose computed tomography or F$cal occult blood test or f$cal immunochemical test or stool tests or smear test or Human papillomavirus test or alphapapillomavirus or papanicolaou test or mammogram, clinical breast examination or breast self-examination or Prostate-specific antigen test or dermoscopy).mp. [mp=title, abstract, original title, name of substance word, subject heading word, floating sub-heading word, keyword heading word, organism supplementary concept word, protocol supplementary concept word, rare disease supplementary concept word, unique identifier, synonyms])

Limit to reviews, humans and English in Ovid MEDLINE

Limit to humans and English in PsychInfo and EMBASE
